# Advanced Digital Workflow for Lateral Orbitotomy in Orbital Dermoid Cysts: Integration of Point-of-Care Manufacturing and Intraoperative Navigation

**DOI:** 10.3390/jcm15030937

**Published:** 2026-01-23

**Authors:** Gonzalo Ruiz-de-Leon, Manuel Tousidonis, Jose-Ignacio Salmeron, Ruben Perez-Mañanes, Sara Alvarez-Mokthari, Marta Benito-Anguita, Borja Gonzalez-Moure, Diego Fernandez-Acosta, Susana Gomez de los Infantes-Peña, Myriam Rodriguez-Rodriguez, Carlota Ortiz-Garcia, Ismael Nieva-Pascual, Pilar Cifuentes-Canorea, Jose-Luis Urcelay, Santiago Ochandiano

**Affiliations:** 1 Department of Oral and Maxillofacial Surgery, Gregorio Marañon University Hospital, 28007 Madrid, Spain; 2 Gregorio Marañón Research Institute, 28007 Madrid, Spain; 3 Advanced Planning and 3D Manufacturing Unit (UPAM3D), Hospital General Universitario Gregorio Marañón, Dr. Esquerdo 46, 28007 Madrid, Spain; 4 Department of Ophthalmology, Gregorio Marañon University Hospital, 28007 Madrid, Spain

**Keywords:** orbitotomy, orbital neoplasms, virtual surgical planning, intraoperative navigation, patient-specific implants, piezoelectric surgery, structured light scanning, three-dimensional printing

## Abstract

**Background:** Orbital dermoid cysts are common benign lesions; however, deep-seated or recurrent lesions near the orbital apex pose major surgical challenges due to their proximity to critical neurovascular structures. Lateral orbitotomy remains the reference approach, but accurate osteotomies and stable reconstruction can be difficult to achieve using conventional techniques. This study reports our initial experience using a fully digital, hospital-based point-of-care (POC) workflow to enhance precision and safety in complex orbital dermoid cyst surgery. **Methods:** We present a case series of three patients with orbital dermoid cysts treated at a tertiary center (2024–2025) using a comprehensive digital workflow. Preoperative assessment included CT and/or MRI followed by virtual surgical planning (VSP) with orbit–tumor segmentation and 3D modeling. Cutting guides and patient-specific implants (PSIs) were manufactured in-house under a certified hospital-based POC protocol. Surgical strategies were tailored to each lesion and included piezoelectric osteotomy, intraoperative navigation, intraoperative CT, and structured-light scanning when indicated. **Results:** Complete en bloc resection was achieved in all cases without capsular rupture or optic nerve injury. Intraoperative CT confirmed complete lesion removal and accurate PSI positioning and fitting. Structured-light scanning enabled radiation-free postoperative monitoring when used. All patients preserved full ocular motility, visual acuity, and facial symmetry, with no complications or recurrences during follow-up. **Conclusions:** The integration of VSP, in-house POC manufacturing, and image-guided surgery within a lateral orbitotomy approach provides a reproducible and fully integrated workflow. This strategy appears to improve surgical precision and safety while supporting optimal long-term functional and aesthetic outcomes in challenging orbital dermoid cyst cases.

## 1. Introduction

Orbital dermoid cysts are congenital tumors that account for approximately 14% of all orbital tumors and 20% of benign orbital tumors, primarily affecting young individuals under the age of 15 [1,2]. They are generally classified as simple, juxtascleral, or soft, and most are superficial, commonly located in the eyebrow region, where diagnosis is usually clinical based on the detection of a mobile mass related to bony structures. Only 0.5% of dermoid cysts are deep and often remain asymptomatic until adolescence [1]. Histologically, they contain hair, keratin, and sebaceous or sweat glands, and imaging with computed tomography (CT) or magnetic resonance imaging (MRI) is essential to define their extent and relationship with adjacent structures [3,4].

The orbital apex represents a particularly challenging region because of the high density of delicate neurovascular structures essential for vision. Lesions in this area may remain clinically silent until they cause compressive symptoms such as optic neuropathy and vision loss, underscoring the need for early diagnosis and surgical management [5]. However, the confined anatomical space of the orbital apex complicates surgical exposure and increases the risk of iatrogenic injury. Ophthalmologists and neurosurgeons have described multiple surgical approaches to improve access, including lateral orbitotomy, extended hemicoronal incisions, and transcranial techniques such as the fronto-orbito-zygomatic approach [6,7]. While these methods provide wide exposure, they are associated with significant drawbacks, including extensive dissection, longer recovery times, and potential morbidity related to neurosurgical maneuvers [5,6,7,8].

Lateral orbitotomy remains the reference approach for accessing the lateral and posterior orbit, offering direct control of the lateral orbital wall and safe entry into retrobulbar spaces [6]. Nevertheless, the procedure requires precise osteotomies and stable replacement of the bone fragments to avoid aesthetic or functional sequelae. In this context, recent technological advances have transformed orbital surgery. Virtual surgical planning (VSP), 3D-printed anatomical models, patient-specific cutting guides, and customized patient-specific implants (PSI) facilitate precise osteotomies and reliable restoration of orbital contour and volume. Complementary technologies such as piezoelectric osteotomy, intraoperative navigation, and intraoperative CT enhance surgical safety in anatomically demanding regions, while structured light scanning provides a radiation-free method for postoperative assessment of orbital symmetry [9,10,11].

The aim of this study is to present a case series of patients with orbital dermoid cysts managed through lateral orbitotomy and to compare different treatment strategies according to lesion location and complexity. We highlight how the integration of VSP, PSI, piezoelectric instrumentation, intraoperative verification, and structured light scanning may contribute to safer, more precise, and reproducible orbital surgery, with improved functional and aesthetic outcomes.

## 2. Materials and Methods

A descriptive case series was conducted including three patients diagnosed with orbital dermoid cysts and treated between 2024 and 2025 at the Hospital General Universitario Gregorio Marañón (Madrid, Spain), a tertiary referral center equipped with an in-house additive manufacturing laboratory (UPAM3D). The primary objective of this study is to describe the technical feasibility and integration of an advanced digital workflow in a clinical setting. Given the descriptive nature of this case series, it is intended to demonstrate the implementation of these technologies rather than to provide a comparative analysis of clinical superiority over traditional techniques.

### 2.1. Patient Selection and Ethical Considerations

Inclusion criteria were patients with deep orbital dermoid cysts requiring lateral orbitotomy, and availability of thin-slice CT scans (<1 mm). Exclusion criteria included a previous history of orbital surgery, prior chemotherapy or radiotherapy in the craniofacial area, and systemic diseases affecting bone healing. Inclusion criteria comprised patients with imaging-confirmed orbital dermoid cysts surgically managed by the multidisciplinary orbital team and with complete clinical documentation. Patients with non-dermoid cystic lesions, incomplete data, or missing consent were excluded. The study was conducted in accordance with the Declaration of Helsinki and reviewed by the Institutional Ethics Committee of the Hospital General Universitario Gregorio Marañón, which waived formal approval due to its retrospective, descriptive nature (protocol code: waived/2024/OMF-POC-001). Written informed consent was obtained from all participants for surgery, data use, and publication of images.

### 2.2. Imaging and Preoperative Evaluation

All patients underwent full ophthalmologic evaluation. Cases 1 and 2 were studied using contrast-enhanced computed tomography (CT) and magnetic resonance imaging (MRI), whereas Case 3 was evaluated using MRI alone due to age (19 years) and previous diagnosis. Imaging data were used to determine lesion size, intra- or extraconal location, and proximity to critical structures.

### 2.3. Virtual Surgical Planning and Additive Manufacturing

Virtual surgical planning (VSP) was a multidisciplinary process. The surgical team identified the osteotomy lines and the desired orbital volume, while biomedical engineers from the Point-of-Care (POC) unit performed the segmentation and CAD design. This collaborative ‘in-house’ approach allowed for real-time adjustments before 3D printing. Virtual surgical planning (VSP) was performed using CT/MRI DICOM datasets for orbit and lesion segmentation. Stereolithographic orbit–tumor models were created to define the osteotomy window and cleavage plane. When required, patient-specific cutting guides were fabricated in biocompatible resin Biomed Clear Resin (Formlabs, Somerville, MA, USA; SLA/DLP printing) or titanium for enhanced rigidity. Patient-specific implants (PSI; Ti-6Al-4V ELI titanium) were designed to restore orbital contour and volume. All models, guides, and implants were produced in-house at UPAM3D under the institutional license (since 2023) for hospital-based point-of-care (POC) production of implantable devices, in compliance with Regulation (EU) 2017/745 (MDR), ISO 13485 (quality management), ISO 14971 (risk management), and ISO 10993 (biocompatibility) [12,13,14,15].

### 2.4. Surgical Procedure

The surgical approach was individualized according to the lesion site and extent: an upper eyelid crease incision (blepharoplasty type) for anterior or superolateral lesions and an extended hemicoronal incision for retroconal or apical lesions. All osteotomies were performed with a piezoelectric device (Mectron S.p.A., Carasco, Italy) to minimize thermal and mechanical trauma. Intraoperative navigation was employed in Case 1, intraoperative CT verification in Case 3, and both bone flaps and PSI were fixed with titanium screws tailored to planned bone thickness. Layered closure was performed in all cases.

### 2.5. Postoperative Care and Follow-Up

Standard postoperative management included prophylactic antibiotics, corticosteroids when indicated, analgesia, and head elevation. Patients were followed clinically for a minimum of six months to assess pain, diplopia, visual acuity, ocular motility, and aesthetic outcome. Control CT scans were obtained when necessary. In Case 3, postoperative structured-light scanning (Artec Eva, Artec 3D, Luxembourg) enabled radiation-free assessment of orbital symmetry and implant integration. Intraoperative structured-light scanning was performed using a handheld scanner. To ensure repeatability, the alignment protocol involved a multi-step registration process using fixed anatomical landmarks (frontal and malar bones). While this technology is innovative, we acknowledge that it remains operator-dependent and its use in this series was exploratory.

### 2.6. Data and Materials Availability

All digital workflows (segmentation, modeling, guide/implant design) were performed using licensed hospital software (Mimics and 3-matic Medical, Materialise NV, Leuven, Belgium; Ultimaker Cura, UltiMaker BV, Utrecht, The Netherlands; Bambu Studio, Bambu Lab, Shenzhen, China; PrusaSlicer, Prusa Research a.s., Prague, Czech Republic; PreForm, Formlabs Inc., Somerville, MA, USA) under controlled conditions at UPAM3D. Data supporting the findings of this study are available from the corresponding author upon reasonable request, in compliance with institutional and patient privacy regulations. During the preparation of this manuscript, the author(s) used ChatGPT (OpenAI, GPT-5.2) for the purpose of language editing and improving clarity and readability of the text. The tool was not used for study design, data collection, analysis, or interpretation of results. The authors have reviewed and edited the output and take full responsibility for the content of this publication.

## 3. Results

### 3.1. Clinical Overview

Three female patients (ages 19, 41, and 47 years) diagnosed with orbital dermoid cysts were treated between 2024 and 2025 at a tertiary referral hospital using an advanced digital workflow integrating virtual surgical planning (VSP), point-of-care (POC) manufacturing, and intraoperative verification technologies. Lesions were primarily located in the superotemporal orbital quadrant, involving both intra- and extraconal spaces. Each case was planned and executed by a multidisciplinary team including Oral and Maxillofacial Surgery and Ophthalmology. Complete en bloc excision was achieved in all patients without capsular rupture or optic nerve injury. Osteotomies were accurate, reconstructions restored orbital contour and volume, and intraoperative verification (navigation or CT) confirmed resection completeness and optimal implant adaptation. Structured-light scanning was used in selected cases for postoperative monitoring of symmetry and implant integration. All patients preserved full ocular motility and visual acuity, with no recurrences or complications during follow-up. Table 1 summarizes demographic, diagnostic, and procedural data.

### 3.2. Clinical Case 1

A 47-year-old woman presented with right-eye (OD) proptosis and periorbital pain for 10 days. No diplopia or acute vision loss was reported. Clinical examination revealed proptosis and pain on dextroversion of the OD; visual acuity (VA): 0.16 in OD and 0.8 in the left eye (OS). Fluorescein staining revealed superficial punctate keratitis, and fundus examination showed an elevated, obliquely oriented optic disc. Urgent orbital computed tomography (CT) demonstrated an intra- and extraconal superotemporal lesion, consistent with a dermoid cyst. Magnetic resonance imaging (MRI) confirmed retroconal localization with a fluid–fluid level, compatible with a cystic lesion near the optic nerve.

Planning and Procedure: Virtual surgical planning was performed using CT and MRI to define the osteotomy trajectory and its proximity to the optic nerve and levator–superior rectus complex. An extended hemicoronal orbitotomy was designed with a quadrangular bone window, and intraoperative navigation (IN) was calibrated for real-time guidance of piezoelectric (PE) osteotomies. A low-profile titanium miniplate was pre-contoured for stable repositioning of the bone segment after resection. The cyst was completely excised en bloc, preserving the capsule. The lateral orbital wall was repositioned and fixed with 5–7 mm titanium screws (≥2 per side). Total surgical time: 5 h and 10 min.

**Figure 1:**
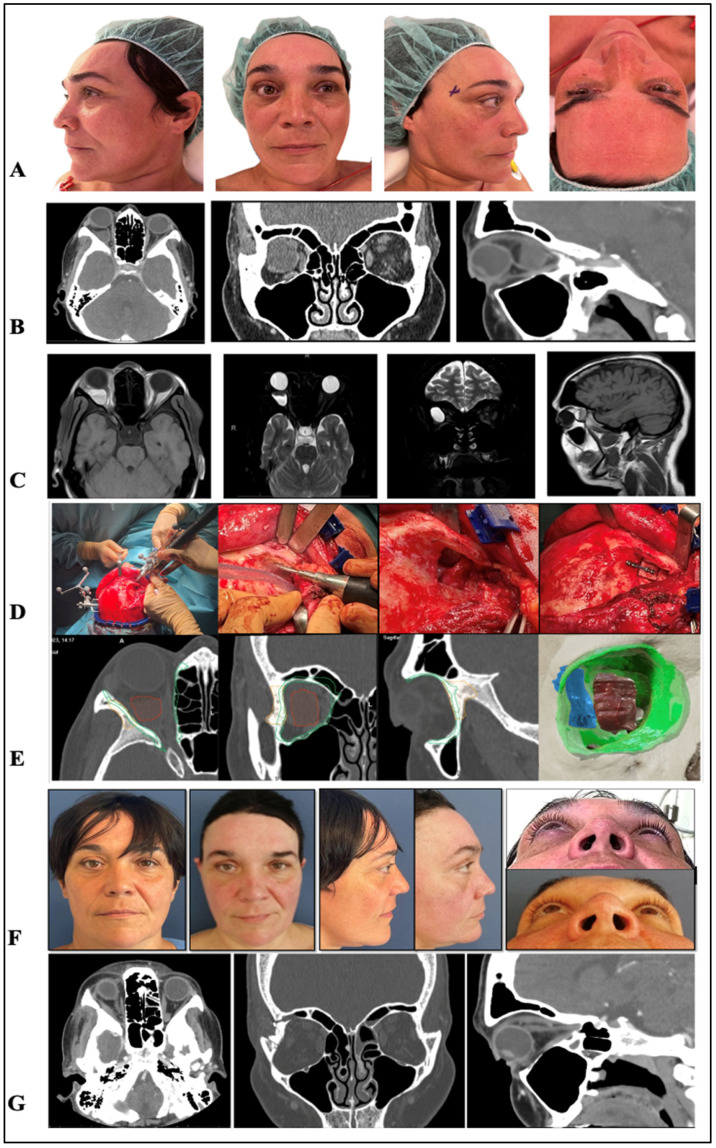
(**A**) Preoperative clinical images showing asymmetry due to right-side proptosis. (**B**,**C**) CT and MRI scans demonstrating a cystic lesion with fluid–fluid level in the superotemporal and retroconal right orbit, particularly evident on T2 sequence. (**D**) Sequential intraoperative views (left to right): navigation sensors placed on the skull and piezoelectric handpiece; planned right lateral orbitotomy guided by navigation; lateral access to the lesion after orbitotomy; and repositioning of the lateral wall with a miniplate and screws. The approach used was an extended hemi-coronal technique, followed by complete cyst resection by Ophthalmology. (**E**) Virtual planning: orbital walls (green), tumor (red), and lateral wall segment for orbitotomy (blue), with axial, coronal, and sagittal CT slices. (**F**) Postoperative clinical images showing correction of asymmetry. (**G**) CT scan at 2 months demonstrating correct repositioning of the lateral wall, restored intraorbital relationships, and correction of exophthalmos, with preserved ocular motility and pupillary reflex despite mild postoperative inflammation.

Outcome: Postoperative management included IV corticosteroids, antibiotics, and analgesia. Recovery was favorable, with resolution of pain and full ocular motility. Visual acuity improved to 0.3 at 1 month. Follow-up CT at 2 months confirmed restored orbital relationships, absence of cyst remnants, and correct bone flap positioning (Figure 1).

Histopathology: Dermoid cyst with keratinaceous material and cutaneous adnexa, without dysplasia or malignancy.

### 3.3. Clinical Case 2

A 41-year-old woman presented with persistent swelling of the left upper eyelid (LUL) for 2 months, without pain or diplopia. Physical examination revealed mild ptosis and S-shaped edema suggestive of lacrimal gland enlargement. Initial management for presumed dacryoadenitis was unsuccessful. CT revealed a 24 × 12 × 28 mm nodular lesion in the left lacrimal gland consistent with dermoid cyst, with rupture and inflammatory extension. MRI confirmed a stable cystic lesion with subcutaneous extension. Surgical intervention was indicated to prevent recurrent inflammation and aesthetic deformity.

Planning and Procedure: A conservative approach was selected due to the superolateral extraconal location and the patient’s age. Virtual surgical planning was performed with orbit–lesion segmentation and 3D stereolithographic modeling. Cutting guides (Biomed Clear Resin, SLA printing) and a customized supraorbital titanium patient-specific implant (PSI) were fabricated in-house (UPAM3D). Surgery was performed via upper blepharoplasty incision under general anesthesia. After precise osteotomy guided by the 3D-printed template, the lesion was resected en bloc and reconstruction performed with the PSI fixed by 7 mm screws. Total surgical time: 3 h and 3 min.

**Figure 2:**
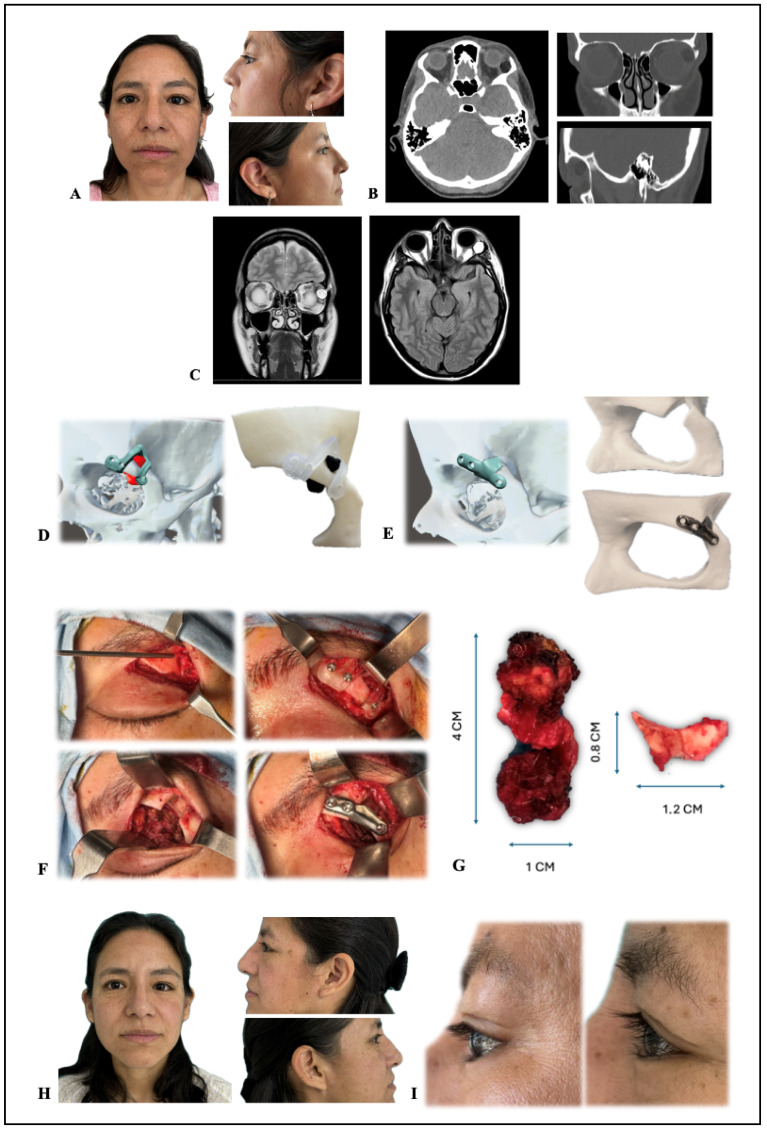
(**A**) Preoperative photos showing the asymmetry caused by the left-side proptosis. (**B**) CT showing a left superotemporal intra and extraconal lesion suggestive of dermoid cyst. (**C**) MRI confirming retroconal localization with cystic component (fluid–fluid level). (**D**) Stereolithographic orbit–tumor models and cutting guides (printed in Biomed Clear Resin). (**E**) PLA models, and a customized supraorbital titanium patient-specific implant (PSI). (**F**) Sequential surgical steps (to be viewed from upper left to right, then lower left to right): (1) upper eyelid crease incision and exposure; (2) fixation of biocompatible resin 3D cutting guide; (3) osteotomy and en bloc resection of the extraconal dermoid cyst; (4) reconstruction with customized supraorbital titanium PSI. (**G**) Osteotomized bone flap from the planned superolateral orbital roof window and microsurgical en bloc resection of the extraconal dermoid cyst. (**H**) Clinical images of the patient at 2-month postoperative follow-up. (**I**) Comparative pre- and 3-month postoperative view of the surgical area.

Outcome: Postoperative course was uneventful, with resolution of edema and restoration of orbital contour. Follow-up CT at 2 months confirmed implant integration and absence of recurrence (Figure 2). Histopathology revealed a ruptured dermoid cyst with chronic inflammation, fibrosis, and foreign-body granulomatous reaction, without malignancy.

### 3.4. Clinical Case 3

A 19-year-old woman was referred for recurrence of an extraconal dermoid cyst in the right orbital roof after prior surgery. Symptoms included blepharitis, intermittent diplopia, and eyelid edema. MRI showed a residual 10 × 11 × 9 mm lesion in the superolateral orbit with bone remodeling but no intraconal extension. A conservative surgical plan was adopted to preserve orbital function and aesthetics.

Planning and Procedure: Virtual planning and segmentation defined the osteotomy margins. Due to prior resin guide fracture in Case 2, a titanium cutting guide and a mesh PSI were designed and fabricated in-house under POC certification. A right upper blepharoplasty incision was used for subperiosteal exposure, followed by piezoelectric osteotomy and en bloc resection. Intraoperative CT confirmed complete removal and accurate PSI adaptation. Fixation was achieved with system 1.5 titanium screws. Total surgical time: 3 h and 5 min.

**Figure 3:**
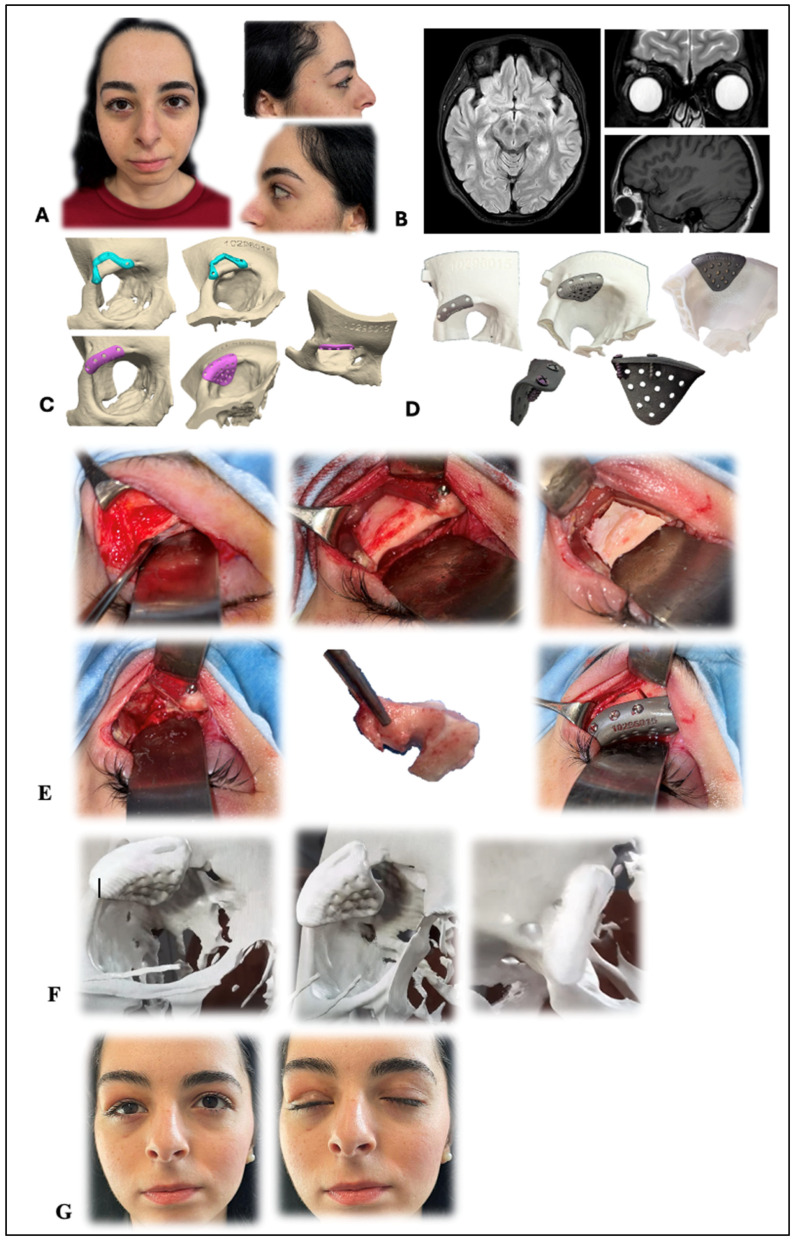
(**A**) Preoperative photos showing the asymmetry caused by the right-side proptosis. (**B**) MRI showing an extraconal dermoid cyst in the right orbit with mild mass effect and bone remodelling. (**C**) Virtual surgical planning with orbit and lesion segmentation for cutting guide and PSI fabrication. (**D**) Titanium cutting guide, PSI, and 3D models—was produced in-house. (**E**) Sequence of images read from right to left and top to bottom: (1) Right upper blepharoplasty approach with subperiosteal exposure. (2) Positioning of titanium cutting guide. (3) Planned osteotomy with piezoelectric device. (4) En bloc resection of extraconal lesion. (5) Resected dermoid cyst. (6) Placement of PSI over repositioned bone flap. (**F**) Intraoperative CT confirming resection, defect adequacy, and PSI placement/fit. (**G**) Clinical images of the patient at 2-month postoperative follow-up.

**Figure 4:**
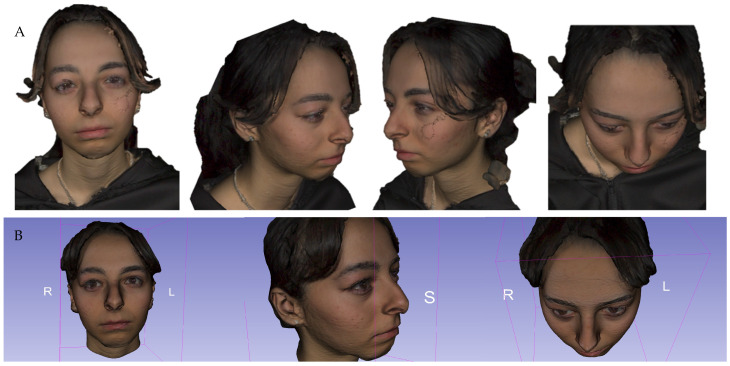
(**A**) Preoperative images used for surgical planning. (**B**) Postoperative follow-up with structured light scanning for assessing implant integration and the stability of the orbital contour.

Outcome: Postoperative recovery was favorable, with complete resolution of diplopia and eyelid swelling (Figure 3). Structured-light scanning verified orbital contour and implant integration without radiation exposure (Figure 4 and Video S1). Histopathology confirmed recurrent dermoid cyst with chronic inflammation, fibrosis, and granulomatous tissue, without malignancy.

### 3.5. Summary of Clinical Outcomes

All three cases achieved complete resection, precise reconstruction, and stable aesthetic and functional outcomes. The use of VSP, 3D cutting guides, and patient-specific implants improved intraoperative precision, while intraoperative navigation, CT, and structured-light scanning enhanced safety and postoperative verification. No recurrences, complications, or vision impairment occurred during follow-up.

Main outcomes include the following:

- Complete en bloc cyst excision with capsule preservation;
- Accurate osteotomies with bone or PSI reconstruction;
- Maintenance of visual acuity and ocular motility;
- Radiological or optical verification of symmetry;
- No postoperative complications during follow-up.

## 4. Discussion

Despite the progressive adoption of minimally invasive and endoscopic techniques, lateral orbitotomy remains the reference approach for lesions located lateral to the optic nerve—both intraconal and extraconal—and for lacrimal gland pathology. It allows direct visualization and control of the lateral wall, provides safe osteotomy lines, and offers favorable access angles to the orbital compartments. Since the classical description by Krönlein, successive modifications have aimed to minimize morbidity and aesthetic sequelae while preserving the fundamental concept of controlled osteotomy and stable bony reconstruction [11,16,17,18].

The choice of surgical access remains crucial to achieving optimal outcomes. The upper eyelid crease incision (blepharoplasty type) is currently preferred for anterior or superolateral lesions because it provides excellent cosmetic camouflage and direct visualization of the orbital roof. However, poor handling of tissue planes may lead to levator injury, retraction, or persistent edema. Conversely, the extended hemicoronal approach allows wide exposure of retroconal and apical regions through an avascular plane between the deep temporal and temporoparietal fasciae; its main limitation—potential injury to the frontal branch of the facial nerve—can be mitigated with careful dissection. In this series, selection between these two incisions was individualized according to lesion depth, location, and cosmetic expectations, in agreement with contemporary literature [11,16,17,18,19,20,21].

To rationalize decision-making, we established a surgical algorithm (Table 2) integrating lesion location (lateral/medial; anterior/apical), vascularity, cleavage plane, proximity to the optic canal, and aesthetic priorities. This systematic approach helps standardize the indication for upper blepharoplasty in anterior–superolateral lesions, extended hemicoronal access for retroconal or apical ones, and transconjunctival or transcaruncular incisions for medial or inferior quadrants. Endonasal endoscopic corridors remain reserved for selected medial intraconal tumors, whereas transcranial routes are indicated for extensive apical or intracranial involvement [18,19,22,23,24,25,26,27,28].

From a technological perspective, our results support the integration of virtual surgical planning (VSP), cutting guides, and patient-specific implants (PSIs) into lateral orbitotomy workflows. These tools enhance precision by defining osteotomy geometry, minimizing intraoperative uncertainty, and ensuring predictable bone flap repositioning. Navigation, particularly valuable near the orbital apex or frontal sinus, provides intraoperative spatial verification and can prevent overextension of osteotomies. Intraoperative CT offers a high level of confidence in assessing resection completeness, bone gap correction, and PSI adaptation, while adhering to ALARA principles [26]. A critical aspect of lateral orbitotomy is the risk of an uncontrolled fracture or ‘bad split’, particularly in cases with thick cortical bone or complex anatomical variations. The integration of virtual surgical planning (VSP) and patient-specific cutting guides allows for a precise definition of the osteotomy planes, ensuring that the bone cuts are executed exactly as planned. Furthermore, the use of piezoelectric surgery in combination with these guides enhances depth control and force distribution. This technological synergy minimizes the mechanical stress on the orbital rims, significantly reducing the likelihood of unintended fractures and ensuring a predictable bone segment for subsequent anatomical reconstruction.

Among emerging optical technologies, structured-light surface scanning (SLS) offers a non-invasive, radiation-free alternative for three-dimensional assessment of the orbitopalpebral contour. This method enables submillimetric mapping, facilitating an objective evaluation of symmetry and reconstruction stability while avoiding the cumulative radiation associated with serial CT-based morphometry [27,28,29,30]. In the present workflow, SLS was implemented both intraoperatively—to assist in flap alignment—and postoperatively for aesthetic documentation. However, it is essential to clarify that its role in this series was primarily exploratory and descriptive. While SLS provides high-resolution surface data, it served to complement surgical documentation rather than as a validated quantitative metric. Nevertheless, its high reproducibility and ease of acquisition suggest significant potential for future standardized follow-up protocols, where optical and radiological data could be integrated through vector-based analyses to provide more robust quantitative outcomes.

Piezoelectric osteotomy, also incorporated into our workflow, provided precise bone cuts with minimal thermal damage and selective preservation of soft tissues. Although slower than rotary instruments and limited by fewer insert geometries, it improved control in proximity to neurovascular structures—an advantage especially relevant for intraconal or apical lesions [12,31,32,33,34,35].

In all three cases, the combined use of tailored access routes and digital technologies enabled accurate osteotomies, complete tumor resection, and stable reconstruction, with operative times ranging from 3 to 5 h and without major complications. These results align with previously published series employing technology-assisted orbital approaches under rigorous anatomical criteria [7,18,19,20,22,23,24,25,26,27,28]. Regarding outcome assessment, although clinical and aesthetic results were favorable, our study lacks standardized quantitative metrics such as validated orbital symmetry analysis or Patient-Reported Outcome Measures (PROMs). Future studies should incorporate these tools to provide more objective data. Furthermore, while the current follow-up was sufficient to observe immediate post-operative recovery, longer-term observation is required to rule out late recurrences and ensure the stability of the PSI.

From a manufacturing and regulatory perspective, our study adds the point-of-care (POC) production dimension, performed under a hospital license aligned with Regulation (EU) 2017/745 (MDR) and the health institution exemption (MDCG 2021-1 Rev.1). Compliance with ISO 13485 (quality management), ISO 14971 (risk management), and ISO 10993 (biocompatibility) ensures full traceability across the digital thread (DICOM → segmentation → design → additive manufacturing → post-processing → sterilization → implantation) [12,13,14,15,36,37,38,39]. This regulated environment minimizes risk, guarantees material conformity (Ti-6Al-4V ELI), and supports clinical auditability. Notably, after a resin guide fracture in Case 2, switching to titanium improved intraoperative robustness and reliability. Economic considerations are paramount for the widespread adoption of these technologies. Although the initial setup of a Point-of-Care (POC) 3D manufacturing unit and the acquisition of navigation systems require a significant investment, the ‘in-house’ model can reduce long-term costs. By eliminating the need for external outsourcing of PSIs—which often involves high costs and long delivery times—hospitals can achieve greater efficiency. Furthermore, the reduction in surgical time and the potentially lower rate of complications and reinterventions contribute to the overall cost-effectiveness of the workflow.

Furthermore, our digital workflow should be contextualized alongside emerging robotic techniques. Robotic-assisted surgery offers high precision and stability in the management of orbital diseases. While our POC approach focuses on customization and cost-effectiveness through PSI and navigation, robotic systems represent a complementary frontier that may further enhance safety in advanced periocular tumor resections.

The implementation of such a digitally integrated, MDR-compliant workflow demonstrates that advanced orbitotomies can be performed safely within a hospital-based production system. However, the small sample size and descriptive nature of our study limit the generalizability of findings. The implementation of this digital workflow involves a non-negligible learning curve. Surgeons must become familiar with virtual planning software and the integration of intraoperative data. This transition is significantly facilitated by the presence of dedicated biomedical engineers within the surgical department. In our experience, after an initial period of interdisciplinary training, the time required for virtual planning and the setup of intraoperative navigation decreases substantially, allowing it to become part of the routine surgical protocol.

It is important to acknowledge that the workflow described herein represents an optimal or ‘best-case’ scenario, supported by the high level of technological integration and multidisciplinary expertise available at a tertiary referral center. We recognize that full implementation—including intraoperative CT and in-house MDR-compliant manufacturing—may not be feasible in all clinical settings. However, this workflow is modular; essential components such as virtual planning and 3D-printed anatomical models can be adopted independently to improve surgical safety even in resource-limited environments, while tools like intraoperative navigation or structured-light scanning provide incremental value for complex cases.

To advance clinical evidence, future multicenter studies and prospective clinical trials are warranted to compare digitally assisted versus conventional orbitotomy in terms of accuracy, operative efficiency, functional recovery, aesthetic results, and cost-effectiveness. Moreover, the development of standardized structured-light scanning protocols—integrating defined landmarks, tolerance thresholds (<1–2 mm asymmetry), and cross-validation against CT data—could facilitate multicenter reproducibility and objective benchmarking of outcomes [40,41,42,43,44].

Ultimately, the combination of VSP, POC manufacturing, intraoperative verification, and structured-light evaluation represents a paradigm shift toward quantifiable, traceable, and patient-specific orbital surgery. Consolidating this workflow through collaborative registries and harmonized quality indicators may strengthen its clinical adoption and set the foundation for next-generation precision surgery in craniofacial and orbital pathology.

Study Limitations: This report is a pilot case series and should be interpreted as such. The small sample size and the absence of a control group are inherent limitations. Therefore, large-scale prospective studies are necessary to confirm the clinical superiority and long-term stability of these preliminary results.

## 5. Conclusions

Lateral orbitotomy remains the preferred approach for lesions located lateral to the optic nerve, provided that it is tailored to lesion depth, anatomical constraints, aesthetic objectives, and surgeon experience. In this small case series, the integration of virtual surgical planning, patient-specific guides and implants, piezoelectric osteotomy, and selective intraoperative verification (navigation, CT, structured-light scanning) enabled accurate osteotomies, stable orbital reconstruction, and full symptom resolution without major complications or procedural delays.

Hospital-based point-of-care manufacturing performed under MDR-compliant standards proved both feasible and safe, ensuring full traceability and adaptive risk control throughout the workflow. Structured-light scanning offered a reproducible, radiation-free metric for postoperative evaluation of orbital symmetry and implant adaptation. It is important to acknowledge that the described workflow represents an optimal ‘best-case’ scenario, made possible by the integration of multiple advanced technologies in a tertiary referral center. We recognize that the full implementation of POC manufacturing, navigation, and intraoperative CT may not be feasible in all clinical settings. However, the modular nature of this workflow allows other institutions to adopt specific elements—such as virtual planning or 3D-printed anatomical models—depending on their local resources and clinical needs. Although based on a limited sample, these results demonstrate the potential of a digitally integrated, and auditable workflow to enhance safety, precision, and functional–aesthetic outcomes in modern orbital surgery.

In conclusion, the integration of VSP, POC manufacturing, and intraoperative navigation is a feasible and reliable approach for complex lateral orbitotomies. While this pilot series shows promising results, further studies with larger cohorts and standardized metrics are necessary to evaluate its long-term clinical impact.

## Figures and Tables

**Table 1:** Summary of Clinical Cases.

	Case 1	Case 2	Case 3
Sex/age	Female, 47 years	Female, 41 years	Female, 19 years
Symptoms	Proptosis, right-eye pain, decreased visual acuity, nasal visual-field defect	Chronic upper-eyelid swelling, mild ptosis	Blepharitis, right-eye swelling, intermittent diplopia
Diagnosis	Intra- and extraconal dermoid, right orbit	Ruptured extraconal dermoid of the left lacrimal gland	Residual extraconal dermoid of the right orbital roof
Surgical approach	Extended hemicoronal approach	Upper blepharoplasty approach	Upper blepharoplasty approach
Technology used	Intraoperative navigation	Virtual three-dimensional planning and cutting guides	Virtual three-dimensional planning and cutting guides with structured-light verification
Reconstruction	Bone-flap repositioning with low-profile titanium miniplate	Custom patient-specific implant	Custom patient-specific implant
Operative time	5 h 10 min	3 h 3 min	3 h 5 min
Outcome	Resolution of proptosis; improvement in visual acuity	Resolution of eyelid swelling and diplopia	Resolution of eyelid swelling and diplopia

**Table 2:** Decision tree for selecting orbital access based on the location of the orbital lesion.

Orbital Lesion Requiring Surgical Access			
Lateral to Optic Nerve		Medial to Optic Nerve	
Lateral and Anterior (roof/superolateral)	Lateral and Retroconal/Apical	Medial and Anterior (inferior/medial rim)	Medial and Intraconal/Apical
Upper eyelid blepharoplasty (controlled orbitotomy; scar camouflaged)	Extended hemicoronal (±fronto-orbito-zygomatic if extensive/apex)	Transconjunctival/Transcaruncular (esthetic, no cutaneous scar)	Endonasal endoscopic (selected medial intraconal) Transcranial if extensive or intracranial
Decision modifiers: lesion size/vascularity, cleavage plane quality, proximity to optic canal/roof/frontal sinus, aesthetic priority, team expertise. Virtual surgical planning with patient-specific guides and implants (PSI) Navigation for deep/apical windows; intraoperative CT to confirm bony cuts or PSI fit; structured light for contour without radiation Piezoelectric osteotomy for fine kerfs and soft-tissue selectivity			

## Data Availability

The data presented in this study are available on request from the corresponding author.

## Supplementary Materials

The following supporting information can be downloaded at https://www.mdpi.com/article/10.3390/jcm15030937/s1. Video S1: Structured-light 3D surface capture of the craniomaxillofacial region using a medical-grade structured-light scanner; Video S2: Smartphone-based 3D surface capture of the same region using a mobile photogrammetry workflow for comparison.

## Abbreviations

The following abbreviations are used in this manuscript:

3D	Three-Dimensional
ALARA	As Low As Reasonably Achievable
CAPA	Corrective and Preventive Actions
CT	Computed Tomography
CT/MRI	Computed Tomography/Magnetic Resonance Imaging
DICOM	Digital Imaging and Communications in Medicine
DLP	Digital Light Processing
IN	Intraoperative (Infrared) Navigation
ISO	International Organization for Standardization
IV	Intravenous
LUL	Left Upper Eyelid
MDR	Medical Device Regulation (EU 2017/745)
MRI	Magnetic Resonance Imaging
OD	Oculus Dexter (Right Eye)
OS	Oculus Sinister (Left Eye)
PE	Piezoelectric
PLA	Polylactic Acid
POC	Point-of-Care
PSI	Patient-Specific Implant(s)
SLA	Stereolithography
Ti-6Al-4V ELI	Titanium Alloy, Extra-Low Interstitial
UPAM3D	Additive Manufacturing and 3D Printing Unit (Hospital Gregorio Marañón)
VA	Visual Acuity
VSP	Virtual Surgical Planning
